# Post-independence health research productivity in Portuguese-speaking African countries: A bibliometric analysis of 43 years of research and higher education in Mozambique

**DOI:** 10.1016/j.heliyon.2024.e35767

**Published:** 2024-08-03

**Authors:** Assucênio Chissaque, Esperança Guimarães, Cesar H. Limaymanta, Carolina Conjo, Bettencourt Preto Sebastião Capece, Luzia Gonçalves, Nilsa de Deus, Isabel Craveiro

**Affiliations:** a Instituto Nacional de Saúde (INS), Marracuene, Mozambique; b Instituto de Higiene e Medicina Tropical (IHMT), Universidade Nova de Lisboa, Portugal; c Departamento de Bibliotecología y Ciencias de la Información, Universidad Nacional Mayor de San Marcos, Peru; d Universidade Zambeze, Matacuane, Beira, Sofala, Mozambique; e Global Health and Tropical Medicine, GHTM, LA-REAL, Instituto de Higiene e Medicina Tropical, IHMT, Universidade NOVA de Lisboa, Portugal; f Centro de Estatística e Aplicações da Universidade de Lisboa, Lisboa, Portugal; g z-Stat4life, Lisbon, Portugal; h Departamento de Ciências Biológicas, Universidade Eduardo Mondlane, Mozambique

**Keywords:** Health science research, Higher education institutions, PALOP, Productivity, Bibliometric, Mozambique

## Abstract

**Background:**

Africa has a high double burden of infectious and non-communicable diseases underscoring the critical need for robust scientific research. However, it is also associated with low scientific research productivity. Mozambique, which gained independence in 1975, serves as a poignant example. However, there remains a notable scarcity of evidence evaluating the country's trajectory in scientific and academic development. This study aims to evaluate 43 years of health-related scientific knowledge production through bibliometric analysis, focusing on key indicators. Additionally, it seeks to characterize the higher education institutions within the country.

**Methods:**

The data was retrieved from the Web of Science Core Collection using an advanced search tool with Boolean research strategies, covering the period from 1976 to 2022 for all PALOP and 1976 to 2019 for Mozambique. To map Mozambican higher institutions, information was sourced from the Ministry of Science Technology and Higher Education database. Descriptive statistics were employed to summarize the findings, while the VOSviewer program version 1.6.19 was utilized to visualize distance-based bibliometric networks, focusing on co-authorship among institutions and keyword co-occurrence.

**Results:**

Portuguese-speaking African countries (PALOP) contribute 2.5 % (10,933 out of 442,309) to Africa's total scientific output, with Mozambique leading at 63.6 % (6,951 publications) followed by Angola at 16.6 % (1,811 publications). All PALOP countries experienced decreased scientific productivity during the third year of the COVID-19 pandemic. In Mozambique, over 70 % (1,710 out of 2,380) of health-related publications from 2011 to 2019 were concentrated in this period. Key journals for health sciences include PLOS ONE, Malaria Journal, and Tropical Medicine & International Health, focusing on HIV, malaria, and tuberculosis. Higher education institutions in Mozambique show regional disparities, with 67.9 % in the South and only 8.9 % in the North, indicating significant inequality in their distribution across the country.

**Conclusion:**

This study highlights Mozambique's significant progress in health research productivity over 43 years, establishing it as a leader among PALOP countries. The substantial increase in publications, particularly after 2008, underscores the nation's growing research capacity and commitment to addressing critical health challenges such as HIV, malaria, and tuberculosis. However, regional disparities in higher education access and limited research contributions from private universities remain high in Mozambique.

## 1 Background

Scientific knowledge production in Africa, particularly in sub-Saharan Africa, is challenging considering the limited financial resources available across continent [1]. While progress has been made in increasing the number of scientific publications, the contribution is still not globally significant [[2], [3], [4], [5]], representing only 1 % of the global scientific production [5].

Health research, especially in low-income countries, ought to ensure that the quality of scientific evidence allows the transformation of research into accessible health technologies and policies based on each country's reality [[6], [7], [8]]. However, research capacity is unevenly distributed in Africa where South Africa, Egypt, and Nigeria have the highest scientific productivity [8,9]. Scientific production in Africa is influenced by political and military instabilities, international collaborations, partnerships in research, and a deficit of critical mass (including limited research skills and English language proficiency) [2,[10], [11], [12]].

Between 2010 and 2015, Mozambique was among the African countries with the lowest number of scientific publications contributing only with 0.45 % to the total number of scientific publications across the continent [1]. Scientific publications were scarce across all research fields, in Mozambique, whereby 63.8 % of all publications were related to health science [9,13]. Notwithstanding, even in this field, only 29.1 % of the internationally published works are cited, which may be related to the low quality of design and statistical factuality of the studies, resulting in non-generalizable outcomes [9]. Moreover, a significant proportion of the studies were published in the Portuguese language, which hampers their visibility and competitiveness. Considering all Portuguese-speaking countries (PALOP), Mozambique is among the most productive regarding scientific publications, but the last decade has harbored significant improvements in PALOP [13]. Despite the high number of scientific publications some of them have not been consistently shared with policymakers or presented in a format that's easily understandable and applicable. As a result, most of them may not have been converted into policy priorities within evidence-based policymaking procedures [14]. Mozambique gained independence from Portuguese colonization in 1975. Since then, the country has faced significant growth challenges but has invested in areas such as the economy, agriculture, health, and education [15]. The government has committed to implementing opportunities for scientists with the potential to leverage scientific research by creating more research institutions such as universities, faculties, institutes, academies, and research centers [15]. Furthermore, the Mozambican *Ministério da Saúde* (Ministry of Health) launched the first National Health Research Agenda, which defines the country's research priorities from 2017 to 2020 to allocate the available funds for research more efficiently, strengthen national institutions in research conduct, and enhance the production of evidence aligned with primary health issues and actually a new agenda was launched which covers the years 2024–2028 [16]. Since the implementation of the first higher education institution, *Universidade Eduardo Mondlane* (Eduardo Mondlane University) in 1962 [17], Mozambique has experienced significant growth in its higher education sector.

Globally and in Mozambique, the promotion of scientific production provides greater availability of evidence to guide decision-making and the formulation of health policies [12,18]. Additionally, identifying the determinants of scientific output enables defining research priorities, maximizing resources, enhancing teaching quality and technology, investing in the health professional's training promoting partnerships and collaborations with developed countries, better work environment for researchers, and dissemination of research results [19,20].

A recent report aimed at Mapping Health Sciences Research and Funding in Angola, Cape Verde, Guinea-Bissau, Mozambique, Saint Tome, and Principe, observed that 16 out of 19 publications in PALOP originate from Mozambique [21]. The primary focus of these publications is on diseases with a high burden in the country, specifically HIV/SIDA and Malaria [22]. Although Mozambique ranks highly among PALOP countries it terms of scientific outputs, it is crucial to understand the organization of research and higher education within the country. This study aims to conduct a bibliometric analysis of key indicators of health-related scientific knowledge production in Mozambique over the past 43 years. The analysis includes the yearly publication count by institution and keyword, co-authorship among institutions, keyword bibliometric network, and an overlay map. Additionally, the study seeks to characterize higher education institutions within Mozambique.

## 2 Methods

### 2.1 Study design

The scientific productivity analysis was based on the Web of Science (WoS), as performed previously [23]. The WoS is an online database created by the Institute for Scientific Information (former ISI Web of Knowledge) and in 2019 was home to >34,000 journals and books with solid coverage in the fields of natural sciences, healthcare, engineering, and computer science [24]. The WoS is relevant for bibliometric analyses by allowing the establishment of relevant bibliometric indicators such as publications, year, institutions, funders, number of citations, research areas, authors' ranking, and others. Specific data of Mozambique was restricted to 43 years after the independence of Mozambique (1976–2019), describing the process of higher education and research evolution through the literature review and the country's scientific health-related productivity based on bibliometric analysis. while data for PALOP was extended to 2022. The onset of the database followed two steps. Initially, to determine scientific productivity in PALOP countries we searched in each country: *Moçambique*/Mozambique*; Angola; Cabo Verde*/Cape Verde; *Guiné-Bissau*/Guinea Bissau; *Guiné Equatorial*/Equatorial Guinea; *São Tomé e Príncipe*/Saint Tome and Principe, all the previously mentioned fields using the advanced search tool Boolean operator OR between 1976 and 2022 through each country's name in both, Portuguese and English languages. The research strategy, structured around specific areas, was developed as follows: “1.217 Parasitology - Malaria, Toxoplasmosis & Coccidiosis or 1.66 HIV or 1.156 Healthcare Policy or 1.194 Tuberculosis & Leprosy or 1.23 Antibiotics & Antimicrobials or 1.104 Virology - General or 1.42 Bacteriology or 1.163 Parasitology - General or 1.44 Nutrition & Dietetics or 1.72 Obstetrics & Gynecology or 1.228 Virology - Tropical Diseases or 6.153 Climate Change or 1.55 Urology & Nephrology - General or 1.246 Diarrheal Diseases or 6.146 Anthropology or 1.65 Allergy or 1.37 Cardiology - General or 3.51 Dairy & Animal Sciences or 1.248 Sexually Transmitted Infections or 3.64 Phylogenetics & Genomics or 3.32 Entomology or 1.258 Zoonotic Diseases or 1.179 Oncology or 1.261 Parasitology - Trypanosoma & Leishmania or 6.10 Economics or 1.134 Trauma & Emergency Surgery or 3.180 Microbial Biotechnology or 1.125 Hepatitis or 1.14 Nursing or 3.85 Food Science & Technology”. Finally, we focused on publications from Mozambique excluding meeting abstracts, conference papers, notes, erratum, retracted articles and bibliographic items since the majority are not submitted to peer review or are no longer available for use. Likewise, we excluded articles from other fields outside of health sciences considering that the focus of this paper is to analyze the health science productivity and scientific papers that referred to Mozambique as part of a group of countries (for example articles from Burden of Diseases Group and others which analyzed the global trends of diseases, where Mozambique is referred as a country and not a local study).

Information and data related to higher education were obtained through the Mozambican Ministry of Science, Technology and Higher Education (*Ministério de Ciência, Tecnologia e Ensino Superior*) through a solicitation to the National Directorate of Higher Studies (*Direcção Nacional de Ensino Superior)*.

### 2.2 Data management, variables of interest, and sample size

After generating all the indicators through the WoS database, they were exported to an *Excel* spreadsheet where further selection processes were applied with the primary objective of excluding articles unrelated to the health science research field based on topic/abstract and if necessary, reading the full article. Those articles that were unclear whether the study was conducted in Mozambique or there was no full text and abstract available, were excluded from the analysis. Two independent researchers were involved in the data screening and selection for the analysis, after removing the non-eligible manuscripts a clean database was generated. The dataset for PALOP countries analysis was extracted between 1976 and 2022, aiming to evaluate the impact of COVID-19. This review covered all fields resulting in a final sample size of 10,933 documents.

Specific analysis for Mozambique health science was conducted from 1976 to 2019. Initially, there was a total of 4,688 articles from Mozambique in all fields of which 1,563 articles were removed after defining the area of analysis interest (health science), additional 673 articles were removed as a result of being systematic reviews that included Mozambique in a couple of countries (e.g., articles from Global Burden of Disease) and others that were not peer-reviewed (meeting abstracts, letter, correction, note, news item). The remaining 2,452 articles were screened manually by two researchers of which 2,380 filled the inclusion criteria. Overall, 2380 articles were eligible for analyzing health science productivity in Mozambique. The main bibliometric indicators are described in Table 1.

**Table 1:** Description of bibliometric indicators and variables.

Indicators/structural analysis	Description
**Scientific Productivity per year by country**	All authors are affiliated with institutions based in a specific country; therefore, each paper is captured in the year of publication and affiliations (organization name, city, province, state, postal code, country, or territory), which is calculated with the logarithm of the number of publications per year. This information allows the extraction of productivity rate per year and country [25].
**Scientific Productivity based on type of journal**	Web of Science comprises over 4,300 open-access journals within its collection. Each indexed journal contains a set of articles that tracks the journal's performance by the number of published articles and citations per year and their citations [25]. In summary, it reflects the balance between the quantity and quality of publications
**Bibliometric network of co-authorship between institutions**	Measure the proportion of the papers co-authored by individuals from the same or different institutions. Each circle (node) represents an institution with its size reflecting several documents produced by that institution. This analysis allows us to understand the structure and dynamics of collaborative relationships within the scientific community, helping researchers, policymakers, and funding agencies understand the patterns of knowledge production and dissemination across different institutions and disciplines.
**Bibliometric network of keyword co-occurrence and overlap**	This analysis shows the evolution of the most relevant topics according to the average year of publication. Providing information about the interdisciplinary connections, and emerging research trends across different fields and disciplines.
**Scientific collaboration**	The proportion of institutional collaboration is when authors from the same or different institutions collaborate on the same work/manuscript. Nodes represent individual authors or institutions, and edges represent co-authorship relationships.
**Nodes used for network**	Each node represents a keyword, and its size is proportional to the number of occurrences of a word with other keywords. The colors are the diversity of invisible clusters formed by a collaboration between institutions both inside and outside Mozambique.
**Bibliometric network of keyword co-occurrence**	Each node represents a keyword, and its size is proportional to the number of occurrences of a word. Allowing the analysis to uncover hidden relationships among research topics and identify areas for further investigation or collaboration.
**Journal quartiles**	Referees to the division of academic journals into four equal parts based on their impact factor or other citation metrics. For example, Quartile 1 (Q1) has a higher impact than 2,3, and 4 so as the quartile number increases less impact the journal has within its field.

### 2.3 Data analysis

Data were summarized using descriptive statistics in frequencies (in cross tables and graphs). The VOSviewer program version 1.6.19 was used to map the distance-based bibliometric networks according to co-authorship by institutions and keywords co-occurrence. To obtain the visualization map, VOSviewer applies the association force normalization technique [26], then the VOS mapping technique "visualization of similarities" [27], and finally the clustering technique [28].

## 3 Results

### 3.1 Science productivity in PALOP

This chapter includes analyses covering the years 2019–2022 to understand the impact of the COVID-19 pandemic on research productivity in PALOP after 2019.

**Fig. 1:**
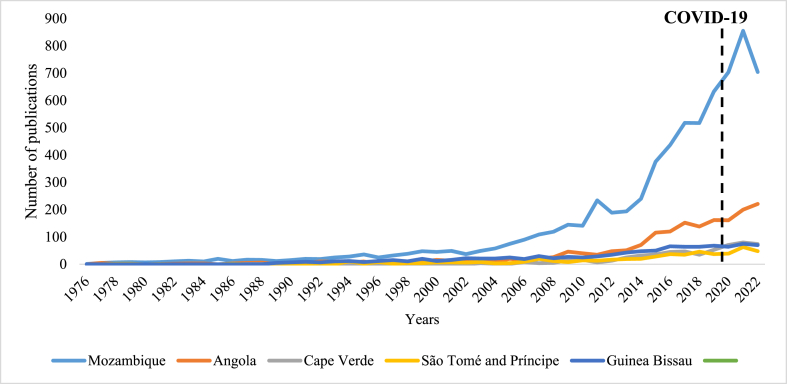
Several scientific articles on WoS in all areas related to the PALOP countries from 1976 to 2019. Important note: The productivity was not divided by country population size.

PALOP contributed only 2.5 % (10,933/442,309) to scientific productivity as measured by the number of published scientific documents in Africa between 1976 and 2022. Among PALOP, Mozambique is the country with the highest scientific production in all research fields and different type of scientific documents, contributing 63.6 % (6,951/10,933), followed by Angola with 16.6 % (1,811/10,933) and Cape Verde with the lowest contribution of 5.4 % (591/10,933) (Fig. 1).

**Impact of COVID-19 on science productivity** **in PALOP (** **2019–2022** **)** **.**

Regarding the impact of COVID-19 on productivity in PALOP, there was a general increase in the number of publications in the second year of the pandemic (2021). However, a decrease in the number of publications was observed in all PALOP, whereby the highest reduction was observed for Mozambique during the third year of the pandemic in 2022 in 151 scientific documents, followed by Angola (21).

### 3.2 Health science scientific productivity in Mozambique (1976–2019)

**Fig. 2:**
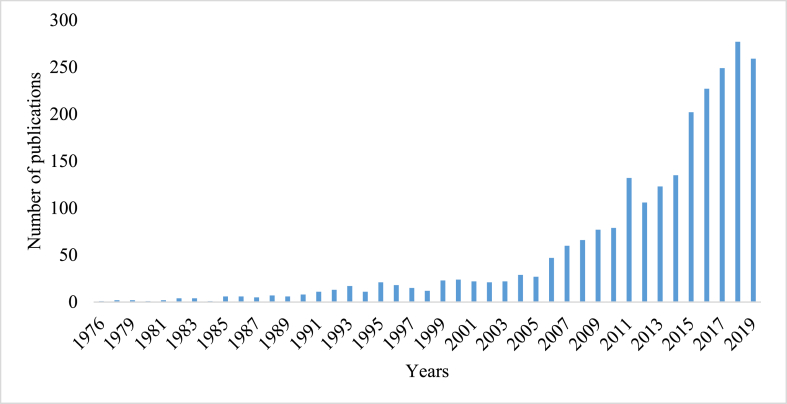
Health scientific production in Mozambique from 1976 to 2019.

This and the following chapters include the years 1976–2019 to exclude bias introduced by the COVID-19 pandemic after 2019. Mozambique published its first health science-related article in 1964, titled “Some Aspects of the Leprosy Problem in the Colony of Mozambique.” The lowest number of publications was observed during the 1970s and 1980s (n = 47), which then tripled in the 1990s (n = 149), showing a continuous increase. A significant milestone was reached in 2011 when annual publications surpassed 100. From 2015 to 2019, annual publications exceeded 200. Notably, the period from 2011 to 2019 accounted for more than 70 % (1,710/2,380) of the total health science output, as shown in Fig. 2.

### 3.3 Journal choice in Mozambique

In this analysis, we have identified 664 journals that published Mozambican health science, using the Scimago Journal & Country Rank based on information in the Scopus® database. Out of 1,924 tracked publications, 7 % are concentrated in PloS One followed by Malaria Journal with 4.4 % and Tropical Medicine & International Health journal with 2.1 %. The remaining journals present a percentage below 2 %. Furthermore, most of the journals listed in the top 20 presented the Q1-Q3 quartile (Table 2).

**Table 2:** Top 20 list of journals where research regarding Mozambique is published, with their absolute and relative frequency and quartile between 1995 and 2019.

Order	Journal	Absolute Frequency	Relative frequency	Quartile
1	PLoS One	134	7.0	Q1
2	Malar. J.	84	4.4	Q1
3	Am. J. Trop. Med. Hyg.	40	2.1	Q2
4	Trop. Med. Int. Health	39	2.0	Q2
5	JAIDS	38	2.0	Q1
6	J. Trop. Pediatr.	34	1.8	Q3
7	Plos Neglect. Trop. Dis.	24	1.2	Q1
8	BMC Public Health	22	1.1	Q1
9	Gynecol.Obstet.Invest.	21	1.1	Q2
10	J. Infect. Dis.	21	1.1	Q1
11	Clin. Infect. Dis.	20	1.0	Q1
12	Trans. Roy. Soc. Trop. Med. Hyg.	20	1.0	Q2
13	Hum. Resour. Health	18	0.9	Q1
14	Lancet	18	0.9	Q1
15	Bull. World Health Organ.	17	0.9	Q1
16	BMC Infect. Dis.	16	0.8	Q1
17	Pediatr. Infect. Dis. J.	16	0.8	Q2
18	PLos Med.	16	0.8	Q1
19	Int. J. Gynecol. Obstet.	15	0.8	Q1
20	AIDS Behav.	14	0.7	Q1

Total of journals: 664, total absolute frequency: 1,924.

### 3.4 Bibliometric network of co-authorship between institutions

**Fig. 3:**
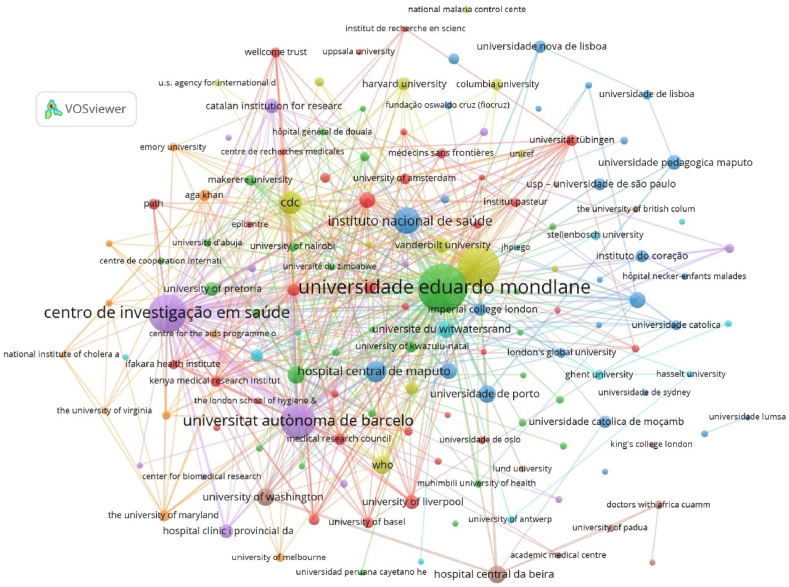
Bibliometric network of co-authorship between institutions. R: −4, A: 2. Threshold: 15. There are 149 institutions.

After normalizing and cleaning the data from the institutions, the bibliometric networks were generated using the VOSviewer program. Fig. 3 presents the co-authorship network among institutions, that contributed to the scientific publications of Mozambique between 1976 and 2019. Each circle (node) represents an institution, and its size is directly proportional to the number of documents from each institution. From a population of 3,040 standardized institutions, those with at least 15 documents were selected, of which 148 meet the threshold. The map illustrates 8 different clusters, each represented by its distinct color. This shows the diversity of invisible clusters formed by collaboration between institutions inside and outside of Mozambique. Among the institutions that stand out in collaborating in Mozambique and globally, the *Ministério da Saúde* had 144 co-authorship links, *Universidade Eduardo Mondlane* with 141 co-authorship links, the *Instituto Nacional de Saúde* (National Institute of Health) with 131 co-authorship links and *Centro de Investigação em Saúde da Manhiça* (Manhiça Health Research Center) with 122 co-authorship links. Among foreign universities that collaborates with Mozambican researchers for publishing the University of Barcelona played an important role with 118 collaboration links.

### 3.5 Ranking of health science productivity in Mozambique by institution and country

*Ministério da Saúde/Instituto Nacional de Saúde*, *Universidade Eduardo Mondlane,Centro de Investigação em Saúde de Manhiça*, and the University of Barcelona are the most productive institutions with 924; 903; 655; 556, and 476 publications related to Mozambican health sciences from 1976 to 2019, respectively. It was also observed that among the countries collaborating with Mozambican Institutions, Spain, the United States, and South Africa are the top 3 (Table 3). It is worth noting that before 2018, the Instituto Nacional de Saúde was considered part of the *Ministério da Saúde*, thus data presented below considers the productivity of both institutions combined. However, an isolated analysis in Supplementary Table 1 shows that the *Instituto Nacional de Saúde* is the 5th best ranked in the country.

**Table 3:** Most productive institutions in health Science Publications in Mozambique (n ≥ 50).

Order	Institution	Fr	Country	Order	Institution	Fr	Country
**1**	*Ministério da Saúde/Instituto Nacional de Saúde (INS)*	924	Mozambique	**19**	University of Pretoria	76	South Africa
**2**	*Universidade Eduardo Mondlane*	903	Mozambique	**20**	*Hospital Clínic I Provincial da Universidade De Barcelona*	74	Spain
**3**	*Centro de Investigação em Saúde de Manhiç*a	556	Mozambique	**21**	University of Liverpool	73	England
**4**	University of Barcelona	476	Spain	**22**	Imperial College London	66	England
**5**	CDC	205	United States	**23**	Harvard University	64	United States
**6**	*Hospital Central de Maputo*	177	Mozambique	**24**	Swiss Tropical and Public Health Institute	62	Switzerland
**7**	University of Cape Town	122	South Africa	**25**	*Universidade Nova de Lisboa	62	Portugal
**8**	World Health Organization	118	World	**26**	Johns Hopkins University	61	United States
**9**	Hospital Central da Beira	118	Mozambique	**27**	*USP – Universidade de São Paulo*	61	Brazil
**10**	University of Washington	116	United States	**28**	*Universidade Católica de Moçambique*	60	Mozambique
**11**	*Universidade de Porto*	115	Portugal	**29**	*Instituto do Coração*	60	Mozambique
**12**	Université du Witwatersrand	110	South Africa	**30**	*Instituto de Higiene e Medicina Tropical	58	Portugal
**13**	Karolinska Institutet	100	Sweden	**31**	Friends Global Health	57	Global
**14**	University of California	99	United States	**32**	Aga Khan	51	Switzerland
**15**	London School of Hygiene & Tropical Medicine	97	England	**33**	Medical Research Council (MRC)	51	England
**16**	*Universidade Pedagógica de Maputo*	86	Mozambique	**34**	Universität Tübingen	50	Germany
**17**	Catalan Institution for Research and Advanced Studies	80	Spain	**35**	University of Kwazulu-Natal	50	South Africa
**18**	Vanderbilt University	78	United States				

**Note:** Fr = Absolute Frequency; *Universidade Nova de Lisboa and Instituto de Higiene e Medicina Tropical were at some point operating independently, for this reason they are not represented in an aggregated manner.

### 3.6 Visualization of the keyword bibliometric network and overlay map

**Fig. 4:**
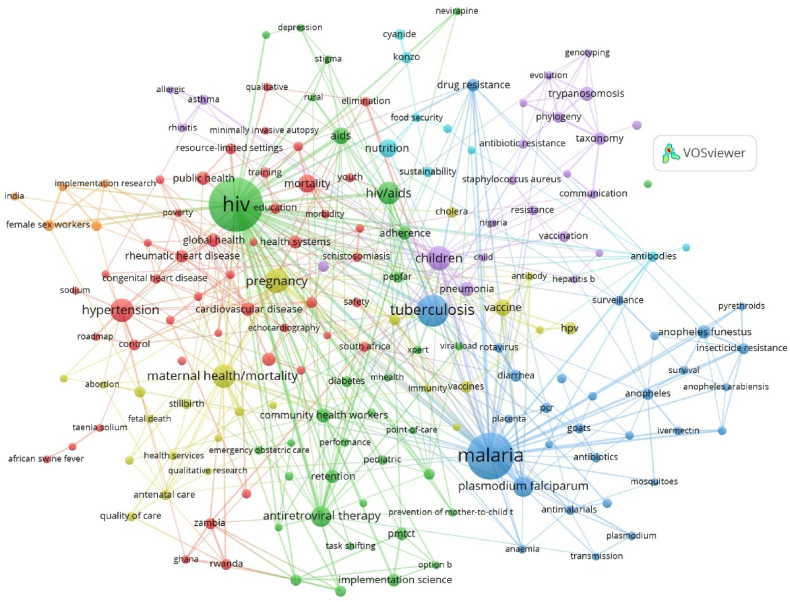
Bibliometric network of keyword co-occurrence. R: −3, A: 1. RA: 0.5, threshold: 5, keywords 172.

Each node in the network represents a keyword, with its size proportional to the number of occurrences alongside other keywords (Fig. 4). The bibliometric network comprises seven clusters, each represented by a different color. These clusters group together keywords that are strongly related based on co-occurrence. The closer two keywords are in the display, the stronger their relationship. Therefore, nodes located close to each other tend to appear in the same field or topic. For instance, "malaria" is closely related to "*Plasmodium falciparum*," and "children" is closely related to "pneumonia."

Table 4 presents an analysis of terms based on their frequency of appearance in the network. Only those with more than 10 occurrences were considered, resulting in 38 keywords meeting this threshold. The top 5 keywords with the highest co-occurrence are HIV, malaria, tuberculosis, children, and pregnancy, with 213, 164, 76, 44, and 43 occurrences, respectively. These keywords predominantly relate to the health sciences, suggesting that this is the primary subject field for publications in Mozambique.

**Table 4:** Most frequent keywords (n ≥ 10).

Order	Keyword	Fr	Order	Keyword	Fr
**1**	HIV	273	**20**	Heart Failure	14
**2**	Malaria	164	**21**	Retention	14
**3**	Tuberculosis	76	**22**	Adherence	13
**4**	Children	44	**23**	*Anopheles funestus*	13
**5**	Pregnancy	43	**24**	Public Health	13
**6**	Maternal Health/Mortality	42	**25**	Drug Resistance	12
**7**	Hypertension	41	**26**	Health Systems	12
**8**	HIV/Aids	39	**27**	HPV	12
**9**	Antiretroviral Therapy	31	**28**	Implementation Science	12
**10**	*Plasmodium Falciparum*	31	**29**	PMTCT	12
**11**	Nutrition	25	**30**	Rheumatic Heart Disease	12
**12**	Mortality	24	**31**	Taxonomy	12
**13**	AIDS	21	**32**	Diarrhea	11
**14**	Vaccine	21	**33**	Vaccines	11
**15**	Pneumonia	16	**34**	Cytokines	10
**16**	Trypanosomosis	16	**35**	Endomyocardial Fibrosis	10
**17**	Community Health Workers	15	**36**	Insecticide Resistance	10
**18**	Cardiovascular Disease	14	**37**	PEPFAR	10
**19**	Global Health	14	**38**	Quality Improvement	10

**Note:** Fr = Absolute frequency.

### 3.7 Keywords overlay map between 2010 and 2019 as average publication year

**Fig. 5:**
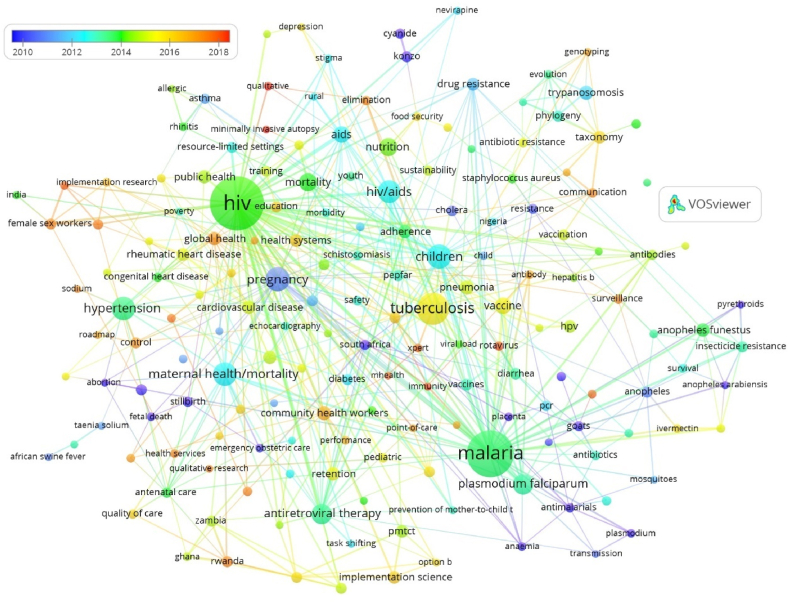
Keywords overlay bibliometric network R: −3, A: 1. RA: 0.5, threshold: 5, total keywords 172.

As a complementary analysis, Fig. 5 presents an overlay map, illustrating the evolution of significant topics within the scientific production of Mozambique, according to the average year of publication. The most frequent keywords in articles published around 2010 to 2012 are displayed in purple and light blue, including terms such as pregnancy, anemia, *Plasmodium*, and cholera. Keywords from articles published on average in 2014–2015 appear in green and yellow, featuring terms like HIV, malaria, Plasmodium falciparum, hypertension, and maternal death. Finally, keywords from articles published between 2017 and 2018 are shown in orange and red. Notably, during this period, the range of topics expanded, and new topics unrelated to infectious diseases began to emerge, including communication, surveillance, global health, and health systems.

### 3.8 Evolution of higher education in Mozambique

**Fig. 6:**
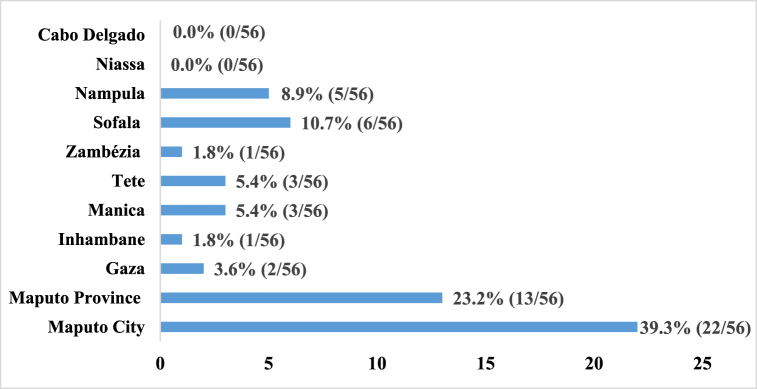
Distribution of higher education institutes public and private by province in Mozambique.

According to Fig. 6, 39.3 % (22/56) of higher education institutions (both public and private) are concentrated in Maputo City, located in the southern region of the country with 67.9 % (38/56). The massive introduction of higher education institutions started in 2005, but the provinces of Inhambane, Zambézia, Niassa, and Cabo Delgado have few public higher educational institutions indicating an uneven distribution across provinces in Mozambique. The distribution of private higher education institutions can be found in Supplementary Table 2.

The *Universidade Eduardo Mondlane* established in 1962 represents the first public University in Mozambique, and Universidade Pedagógica de Maputo in 1985 (23 years later) while the Academia de Ciências Policiais was established 37 years later in 1999. Despite the gap between the establishment of these institutes, they contributed significantly toward human resource training from the 1960s to the 1990s. Later, other higher education institutes were implemented in the country (Table 5).

**Table 5:** The evolution of public universities over time and by the province in Mozambique. Evolution of public higher education institutions from 1976 to 2019 and their distribution by province in Mozambique.

Order	University/institution	Abbreviation	Province	Year^a^
1	Universidade Eduardo Mondlane	UEM	Maputo City	1962
2	Universidade Pedagógica de Maputo	UP	Maputo City	1985
3	Universidade Joaquim Chissano	UJC	Maputo City	1986
4	Academia de Ciências Policiais	ACIPOL	Maputo City	1999
5	Academia Militar	AM	Nampula	2003
6	Instituto Superior de Ciências de Saúde	ISCISA	Maputo City	2003
7	Escola Superior de Ciências Náuticas	ESCN	Maputo City	2004
8	Instituto Superior Politécnico de Gaza	ISPG	Gaza	2005
9	Instituto Superior Politécnico de Manica	ISPM	Manica	2005
10	Instituto Superior Politécnico de Tete	ISPT	Tete	2005
11	Instituto Superior de Contabilidade e Auditoria de Moçambique	ISCAM	Maputo	2005
12	Universidade Lúrio	UniLúrio	Nampula	2006
13	Universidade Zambeze	UniZambeze	Sofala	2006
14	Instituto Superior de Artes e Cultura	ISArC	Maputo Province	2008
15	Instituto Superior Politécnico de Songo	ISPS	Tete	2008
16	Escola Superior de Jornalismo	ESJ	Maputo City	2008
17	Instituto Superior de Estudos de Defesa	ISEDEF	Maputo Province	2011
18	Academia de Altos Estudos Estratégicos	AAEE	Maputo Province	2016
19	Universidade Púngué	Unipungue	Manica	2019
20	Universidade Licungo	UniLicungo	Zambézia	2019
21	Universidade Rovuma	UniRovuma	Nampula	2019
22	Universidade Save	UniSave	Gaza	2019

a Year of creation. **One important note:** there were new delegations and universities created in the last years, however, this is not available on the list of *Direcção Nacional do Ensino Superior,* e.g., in 2019 UP was divided into 5 main universities that are distributed in the three regions of the country namely: Universidade Rovuma, in Nampula province, which cover Niassa and Cabo Delgado former delegation of UP; Universidade Licungo in Quelimane Zambézia province, which includes Beira delegation; Universidade Púnguè in Manica province, which includes Tete province delegation; Universidade Save in Inhambane province, which include Gaza province delegation, and Universidade Pedagógica de Maputo in Maputo city.

### 3.9 Distribution of the higher education institutes by regions and provinces

**Fig. 7:**
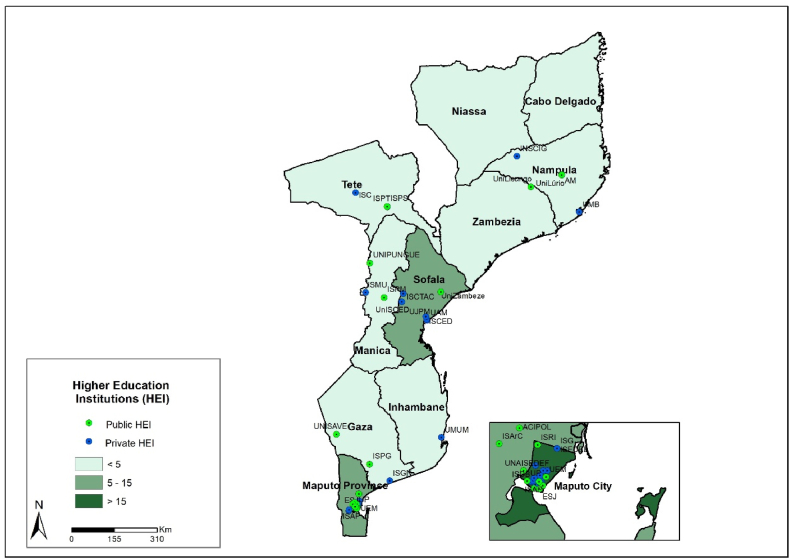
The distribution of private and public higher education institutes in Mozambique.

Fig. 7 shows the distribution of private and public higher education institutions across Mozambique. There is great inequality in the distribution of higher education institutions, with a high concentration in the southern region (Maputo City and province, Gaza, and Inhambane) with 67.9 % (38/56), followed by the central region (Sofala, Manica, Tete, Zambézia) with 23.2 % (13/56) of the higher education institutions located in the northern region (Nampula, Cabo Delgado, Niassa), only 8.9 % (5/56).

## 4 Discussion

Mozambique stands out with the highest scientific productivity among PALOP accounting for 50.2 % of the total of all scientific documents according to Scimago Journal & Country Rank according to the International Science Ranking. However, on a broader African scale, it ranks 25th in terms of significant science productivity, with South Africa, Egypt, and Nigeria leading the pack, according to Scimago Journal & Country Rank according to the International Science Ranking [9]. Our internal data confirms that 63.6 % of scientific production originates from Mozambique among the PALOP. This notable output can be attributed to the country's higher proportion of researchers per million inhabitants with 90 researchers compared to Angola's 49 per million inhabitants [21]. This emphasis on human resource development underscores Mozambique's commitment to fostering research and education, despite enduring a challenging history marked by independence in 1975 and a subsequent 16-year civil war. Nevertheless, the government successfully facilitated the transition from the colonial to the Mozambican education system during the 80s–90s (Law nº 4/83 of March 23, 1983). Additionally, the first constitution (Law nº 4/83 by the approval of Law nº 6/92), placed education, training, and research as priorities [15]. The establishment of the *Universidade Eduardo Mondlane* soon after independence paved the way for educational advancements, with subsequent expansions of higher education institutions across different regions in the early 2000s [29]. Notably, Mozambique has been the sole PALOP country with an operational National Research Fund, the "*Fundo Nacional de* *Investigação*," since 2005. While Angola, gained independence in the same year as Mozambique, the country was devastated by a 27-year-long civil war, which held back the country in all spheres of development including education and research [30]. However, despite these setbacks, Angola has emerged as the second-highest scientific producer among PALOP, attributed partly to investments in higher education, including the establishment of the first public university, Universidade Agostinho Neto [31]. Similar challenges have affected Guinea-Bissau, marked by political unrest and delayed investments in higher education until the early 2000s, hindering its research capabilities [32]. This country shows low investment in educational growth and a delay in implementing higher education and low investment in educational growth. The first universities only appeared in the years 2003/2004, and only then the country developed a strong European and American collaboration to support the training of students [32]. Cape Verde and São Tomé and Príncipe, though spared from civil wars, also faced their obstacles in establishing higher education institutions, with Cape Verde seeing its first university established in 2006 and São Tomé and Príncipe elevating its sole public institution to university status in 2014 [[32], [33], [34]]. Cape Verde and São Tome & Principe must be analyzed thoroughly as they are small countries and the number of students willing to be formed in the country may influence the establishment of new universities or higher education Institutes. This analysis should be interpreted carefully as the size of populations may affect the comparison of productivity between them. PALOP present important asymmetries in health research production, highlighting the need for specialized researchers, targeted funding, and financial programs to enhance competitiveness and facilitate successful grant applications for science and technology development.

All PALOP countries experienced a surge in publications during the second year of the COVID-19 pandemic (2021), followed by a decrease in the third year (2022). Mozambique and Angola were notably affected, with Mozambique witnessing a decrease of 151 scientific documents compared to Angola's with 21. This reduction was expected as researchers focused on responding to the pandemic across various fields. Furthermore, a recent study observed a decrease in the production of non-COVID-19 scientific documents and an increase in COVID-19 ones [35], thus affecting the number of scientific documents as low-income countries took a considerable time to publish data related to COVID-19. Further research is needed to assess the pandemic's impact on scientific production and whether major funders diverted resources to COVID-19 research, potentially neglecting other critical projects, especially in countries with limited independent funding for science.

From 1976 to 2019, Mozambique recorded a total of 2380 publications in the field of health science, with a notable and sustained increase beginning around 2015. Impressively, the subsequent years accounted for 70 % of all publications. This surge in scientific output can be attributed partly to the prominence of key health programs tackling malaria, HIV, and tuberculosis, alongside the strengthening of post-graduate training initiatives at institutions like the *Centro de Investigação em Saúde de Manhiça, Instituto Nacional de Saúde*, and other educational establishments. The collaboration between *Instituto* *Nacional de Saúde* and FIOCRUZ from Brazil has significantly strengthened the capacity of Instituto Nacional de Saúde and the overall health system. This partnership has positively impacted health productivity through postgraduate education, capacity building, and other initiatives.

This exponential trend was also reported in health sciences research productivity in a study conducted in Angola where a four-fold increase was observed between 2004 and 2013 (n = 232) compared to 1979–2003 where only 62 publications were found [35]. We identified 664 different journals publishing Mozambican Health Scientific works, with Plos One and Malaria Journal leading the way at 7.0 % and 4.4 % respectively, mirroring a similar trend in Angola [36]. Notably, the top 10 journals highlighted in our study are open access and predominantly classified in quartile 1, indicating a preference among authors for reputable publications in the field of health sciences. Many of these journals offer full waivers for authors from low-income countries, facilitating greater accessibility to scientific discourse. However, financial constraints remain a hurdle for publication in journals without such waivers, underscoring the need for broader accessibility initiatives.

The *Ministério da Saúde/Instituto Nacional de Saúde, Universidade Eduardo Mondlane, Centro de Investigação em Saúde de Manhiça,* and the University of Barcelona emerged as the primary institutions driving scientific production. The *Instituto Nacional de Saúde's* comprehensive training programs have empowered numerous collaborators, contributing significantly to the volume of published papers. Over the past two decades, a welcome shift has occurred within *Ministério da Saúde*, expanding beyond solely medical professionals to encompass a diverse range of experts such as biologists, pharmacists, and veterinarians. This inclusivity has undoubtedly enriched scientific publications and fostered interdisciplinary collaboration.

Since 2018, the *Instituto Nacional de Saúde* has undergone a significant transition, marked by a new organic status (Decreto-57-2017-Redefinição-INS) [37], granting autonomy to its scientific activities. This shift has allowed researchers from INS to put their affiliation independently of the *Ministério da Saúde.*

Established in 1955 as the Institute of Medical Research of Mozambique (IIMM), under the supervision of the *Instituto de Higiene e Medicina Tropical*, *Instituto Nacional de Saúde* initially focused on tropical disease research and utilized platforms like the Revista Moçambicana de Ciências de Saúde to disseminate Mozambique's health research results. In 1975, it was rebranded as the *Instituto Nacional de Saúde* under Portaria nº 41/75 of 30th August [38]. By 1983, INS assumed the responsibility of publishing the Revista Moçambicana de Ciências de Saúde and assumed a pivotal role in shaping health policies through research and implementation. Throughout the 1990s, INS made significant strides, establishing a research unit in Health Systems and promoting scientific methodology. In 2004, its status was redefined under the Organic Statute, placing it subordinate to the *Ministério da Saúde* and assigning it key responsibilities such as coordinating the National Health Research Agenda and health research (*Diploma Ministeria*l n° 89/2004) [39]. By 2010, INS had formulated its first strategic plan (2010–2014), with a mission to generate evidence for health policy formulation and enhance human resource capacity through postgraduate courses [40]. The approval of the National Health Research Agenda in 2014 further solidified INS's role in guiding health research priorities in Mozambique [40].

Although INS lost its affiliation with *Ministério da Saúde* before the new organic status, it could stand out among institutions and was ranked as the 4th institution with major scientific productivity, which shows the relevant role of this institution for scientific productivity in Mozambique.

Besides *Ministério da Saúde/Instituto Nacional de Saúde, Universidade Eduardo Mondlane* is the second institution with the highest scientific publications and the first higher education institution in Mozambique. The university has benefited from collaborations, notably with Swedish funding through the SIDA/SAREC program in 1978 focused on social sciences and after expanded to natural sciences and scholarship, resulting in an increase in doctoral teachers from 68 in 1990 to 110 in 2002 [41]. Another significant project, *Programa de Desenvolvimento em Saúde Reprodutiva, HIV/SIDA e Assuntos de Família através da Investigação Multidisciplinar Inter-universitária* (DESAFIO), supported by the Belgian government, has been implemented since 2008, encompassing postgraduate programs, scientific research, and university extension. It is also important to highlight the cooperation of Universidade Eduardo Mondlane Italian University that started in 1977. This support started with sending Italian Professors to Mozambique and later on, the focus increased to the training of Universidade Eduardo Mondlane lecturers and researchers and the introduction of advanced technologies and another initiative that resulted in the promotion of One Health approach, where healthy environments, animals, plants, and humans together contribute to the well-being of the planet [42]. The *Centro de Investigação em Saúde de Manhiça*, established in 1996 through collaboration between the governments of Mozambique and Spain, is a vital health research institution focusing on prevalent communicable diseases such as malaria, HIV/AIDS, tuberculosis, and bacterial diseases. Its formation led to the creation of the Manhiça Foundation in 2008, ensuring stable funding for its operations [43]. It later led to the creation of the Manhiça Foundation (2008) and the establishment of a governance model that allows for stable funding to the *Centro de Investigação em Saúde de Manhiça*. Every year, it gives money to the institution through funds that cover the center's basic expenses. This agreement includes the collaboration of ISGlobal Barcelona, a former partner of *Centro de Investigação em Saúde de Manhiça* [44]. This also justifies Mozambique's strong collaboration with Spanish institutions like the University of Barcelona. Also received support from *Universidade Eduardo Mondlane* and *Ministério da Saúde* which are public institutions that impacted scientific productivity through scientific collaboration. Among the top three institutions, *Ministério da Saúde/Instituto Nacional de Saúde* and *Universidade Eduardo Mondlane* present high co-authorship links with different institutions. In general, Spain and the United States of America present a significant contribution to health research in Mozambique. The United States has had diplomatic relations since the year of the proclamation of independence (1975); Mozambique has benefited from this partnership's assistance in different areas mainly the health one, and the great program to highlight is the USA President's Emergency Plan for AIDS Relief (PEPFAR), which has supported the country in counseling, testing and treating HIV and tuberculosis, preventing mother-to-child transmission of HIV and supporting health systems [45], which allowed not only to save Mozambican lives but also to increase scientific production in the country, especially in this field. However, other American organizations such as the Centers for Disease Control and Prevention (CDC), Clinton Foundation, Ariel Glaser Foundation, Elisabeth Glaser Pediatric AIDS Foundation and other partners, also played an important role and established important partnerships with *Universidade Eduardo Mondlane* (Faculty of Medicine), the *Instituto Nacional de Saúde, Ministério da Saúde,* and others [46,47].

Our analysis yielded different results compared to a study conducted in Angola, where most first authors were affiliated with Portuguese institutions. This disparity could be due to the use of different electronic databases; the Angola study relied on Pubmed, while we utilized the Web of Science, known for its stringent journal selection criteria, which may have excluded non-English language articles. This could have implications for collaborations between the two countries in the field of health sciences [36]. Examining institutional collaborations, we observe persistent weak cooperation among African institutions, raising concerns about the prevalence of scientific and technological partnerships within the continent.

The prevalent topics/keywords in Mozambique's scientific research reflect the country's health priorities and the funding received from donnors/funders which emphasize key areas such as HIV, Malaria, and Tuberculosis emerging as key focus areas due to their high national prevalence rates (12.5 %, 39 %, and 361 cases per 100,000 people respectively) [[48], [49], [50], [51]]. Collaborations between Mozambique the United States of America and European countries have played a significant role, particularly in infectious diseases like HIV and tuberculosis. Meanwhile, the emphasis on tropical diseases such as malaria underscores the impact of funding on the research agenda, with substantial scientific documentation in this field [52]. However, it is crucial to expand research efforts to include zoonotic diseases and climate change by adopting a One Health approach. This approach considers the interconnectedness of vector-borne diseases, food security, nutrition, and the potential for future pandemics linked to zoonotic diseases. Consequently, a multisectoral response is necessary to mitigate emerging health threats effectively.

In general, higher education took a long time to gain visibility and expand in the PALOP countries and Mozambique seems to be the country that best organized itself after independence. The government took a big step forward in the first republic in 1983 when the national education system was created allowing the transition from the colonial to the Mozambican system (Law nº 4/83 of March 23, 1983). The second important moment in education took place in 1992 with the implementation of the first constitution of the republic (Law nº 4/83 through the approval of Law nº 6/92) which had as one of the objectives the revision of the law (Law nº 4/83 of March 23, 1983) for the need to train quality scientists capable of leveraging scientific research in Mozambique. Thus, allowing the inception of more research institutions such as Universities, Research Centers, and higher education institutions [14].

Despite their pivotal role in research, universities in Mozambique, particularly those offering Medicine and Health Science courses, contribute minimally to research. *Universidade Eduardo Mondlane* , the country's oldest and largest, was a pioneer in Mozambican research. However, the second public university, Universidade Pedagógica de Moçambique, was established 23 years later, resulting in significant educational disparities and regional inequalities. This 23-year gap impeded the development of a robust academic and research culture across the country, limiting opportunities for higher education and scientific advancement. While the number of higher education institutions has grown, with 72.2 % (16 out of 22) introduced in the last two decades, most are concentrated in the Southern region. This uneven distribution impacts scientific production and forces students to migrate southward for education.

The limited contribution of the higher education in scientific production may be related to i) l limited resources funding for research is often inadequate, leading to a lack of essential resources such as laboratories, laboratory material and equipments, and trained personnel, ii) inadequate infrastructure particularly in universities outside the capital, exacerbating regional disparities in research output, iii) insufficient research culture in many Mozambican universities, there is an insufficient emphasis on research as a core academic activity, and lack of training and support researchers often lack adequate training and mentorship, which are critical for developing research skills and methodologies [53]. Addressing these issues is essential for enhancing research contributions and fostering socio-economic development.

Private higher education institutions have low scientific production, showing the need to invest in scientific writing, publication, and collaboration with institutions that are scientifically established to share the expertise. This is a concerning result as private universities usually have smaller class sizes and more selective admissions, potentially fostering closer faculty-student collaborations and facilitating research productivity.

### 4.1 Study limitations

This study had some limitations namely the fact that Web of Science may not include all scientific journals, particularly those from non-English-speaking regions as PALOP or smaller publishers. This can lead to a bias towards research published in certain languages and/or regions. Web of Science primarily focuses on journal articles and may not adequately capture other forms of scholarly output, such as books, conference proceedings, or preprints [54].

Despite its limitations, the Web of Science is one of the most comprehensive databases for scholarly research, covering a wide range of disciplines. The majority of journals indexed in Web of Science are peer-reviewed, ensuring a certain level of quality and reliability in the content, which makes it a valuable source of high-quality information for bibliometric analysis. Secondly, as this is the first analysis of health productivity in the country there is a lot of data to be reported that is not possible to share in only one document (article), this includes bibliometric analysis of the first and last author raking in publications from Mozambique, funding and others. Although the limitations, this is one of the few documents in Mozambique that shows the health-related research productivity and higher education evolution, which will be useful to research planning and to give the right direction to the research agenda in the country and resource allocation.

### 4.2 Final remarks

The second National Research Agenda approved for the years 2024–2028 focuses on eight key research priority areas. These include infectious diseases, non-communicable diseases, health system and preventive medicine, pharmacovigilance and rational use of medical drugs, neglected and emergent diseases, climate environment and health, mental health, and violence and trauma. The mid-term and long-term evaluation of this agenda should consider the use of bibliometric parameters which was not done for the first and, consequently, may bias the real situation of the country on the reports. To improve research productivity in Mozambique there is a need to overcome some challenges such as a lack of qualified scientists, particularly those with advanced degrees and specialized expertise, and brain drain, where skilled professionals leave the country for better opportunities abroad, exacerbates this issue. The research collaboration, both within Mozambique and internationally, is often limited. Collaborative efforts can enhance research quality, access to funding, innovation, and knowledge exchange, but the lack of networking and weaknesses in partnerships (e.g. authorship) hinder the research ecosystem in Mozambique. Much of the research output in Mozambique is published in Portuguese, which can limit its visibility and accessibility on the international stage. There may be gaps in policy frameworks and institutional support for research and innovation in Mozambique. Strengthening policy guidance, promoting research-friendly regulations, and fostering a supportive environment for innovation are essential for nurturing a vibrant research ecosystem, which calls researchers, institutions, and the government to invest in research infrastructure, human capital development, collaborative networks, and supportive policies. Finally, universities can play a crucial role in building the research capacity of students, faculty, and staff by providing education and training in research methodologies, data analysis techniques, and academic writing, equipping individuals with the skills needed to conduct high-quality research. Mozambique currently lacks robust platforms and mechanisms for researchers to effectively disseminate their findings to policymakers. This deficiency undermines the crucial role research should play in informing policy formulation and decision-making and shaping research priorities within the country [14]. The absence of such channels hampers evidence-based policymaking and limits the potential for meaningful collaboration between researchers and policymakers to address pressing societal challenges.

## 5 Conclusion

Mozambique stands out as the leader in scientific productivity among PALOP countries, a testament to its strategic investments in higher education and health research, coupled with key international partnerships. The surge in health research, particularly notable between 2008 and 2012, has been sustained with remarkable growth. *Universidade Eduardo Mondlane* and the *Ministério da Saúde* have emerged as pivotal contributors to this success story, driving significant advancements in health productivity. Notably, infectious diseases and tropical medicine have emerged as focal points, underscoring both the country's specialization and the commitment of funders. Despite the challenges it faces, Mozambique stands as a beacon, showcasing how concerted efforts can elevate health research production within PALOP countries.

## Ethics approval and consent to participate

Not applicable.

## Availability of data and materials

The datasets used and analyzed during this study are available in a publicly accessible repository, which can be accessed via https://doi.org/10.5281/zenodo.12632759.

## Funding

No funds, grants, or other support was received.

L. G. was partially supported by the 10.13039/501100019370Foundation for Science and Technology, Portugal, through projects UIDB/Multi/04413/2020 and UIDP/Multi/04413/2020 (GHTM), UIDB/00006/2020 and UIDP/00006/2020 (CEAUL) and I. C. was partially supported by the Foundation for Science and Technology, Portugal, through projects UIDB/Multi/04413/2020 and UIDP/Multi/04413/2020 (GHTM).

## CRediT authorship contribution statement

**Assucênio Chissaque:** Writing – review & editing, Writing – original draft, Visualization, Resources, Project administration, Methodology, Investigation, Formal analysis, Data curation, Conceptualization. **Esperança Guimarães:** Writing – review & editing, Visualization, Validation, Conceptualization. **Cesar H. Limaymanta:** Writing – review & editing, Writing – original draft, Visualization, Validation, Supervision, Methodology, Investigation, Formal analysis, Data curation, Conceptualization. **Carolina Conjo:** Writing – review & editing, Methodology, Investigation, Data curation. **Bettencourt Preto Sebastião Capece:** Writing – review & editing, Visualization, Validation, Data curation. **Luzia Gonçalves:** Writing – review & editing, Supervision, Methodology, Investigation, Data curation, Conceptualization. **Nilsa de Deus:** Writing – review & editing, Validation, Supervision, Methodology, Investigation, Conceptualization. **Isabel Craveiro:** Writing – review & editing, Writing – original draft, Visualization, Validation, Supervision, Methodology, Investigation, Data curation, Conceptualization.

## Declaration of competing interest

The authors declare that they have no known competing financial interests or personal relationships that could have appeared to influence the work reported in this paper.

## References

[1] Sooryamoorthy R. (2018). The production of science in Africa: an analysis of publications in the science disciplines, 2000---2015. Scientometrics.

[2] Tijssen R.J.W. (2007). Africa's contribution to the worldwide research literature: new analytical perspectives, trends, and performance indicators. Scientometrics.

[3] Foundation TE. Africa Doubles Research Output Over Past Decade, Moves Towards a Knowledge-Based Economy [Internet]. [cited 2022 February 17]. Available from: https://www.3blmedia.com/news/africa-doubles-research-output-over-past-decade-moves-towards-knowledge-based-economy.

[4] Nordling L. (2014). Africa science plan attacked. Nature.

[5] Blom A., Lan G., Adil M. (2016). https://openknowledge.worldbank.org/handle/10986/23142.

[6] Lundvall B.-A. (2009). Innovation in Africa - toward a realistic vision. African Journal of Science, Technology, Innovation and Development.

[7] World health report 2013: Research for universal health coverage [Internet]. WHO | Regional Office for Africa. [cited 2022 February 17]. Available from: https://www.afro.who.int/publications/world-health-report-2013-research-universal-health-coverage.

[8] Confraria H., Godinho M.M. (2015). The impact of African science: a bibliometric analysis. Scientometrics.

[9] SJR - International Science Ranking [Internet]. [cited 2022 February 19]. Available from: https://www.scimagojr.com/countryrank.php?region=Africa.

[10] Pouris A., Pouris A. (2009). The state of science and technology in Africa (2000–2004): a scientometric assessment. Scientometrics.

[11] Waast R., Rossi P.-L. (2010). Scientific production in arab countries: a bibliometric perspective. Sci. Technol. Soc..

[12] Amarante V., Burger R., Chelwa G., Cockburn J., Kassouf A., McKay A. (2021). Underrepresentation of developing country researchers in development research. Appl. Econ. Lett..

[13] Carvalho PS de, Antunes M. da L. (2018). Retrato da produção científica dos países da Comunidade de Países de Língua Portuguesa (CPLP) em ciências da saúde. XIII Jornadas APDIS, 14-16 março 2018.

[14] Cambe M.I., Botão C., Dulá J., Muamine E., Mahumane S., Alberto C. (2022). The use of research for health systems policy development and implementation in Mozambique: a descriptive study. Glob. Health Sci. Pract..

[15] Matiquite P.C.S. (2019). Ensino superior E pesquisa científica em moçambique. Cadernos de África Contemporânea [Internet].

[16] Agenda-de-Investigação-em-Saúde-Humana-1.pdf [Internet]. [cited 2024 July 17]. Available from: https://ins.gov.mz/wp-content/uploads/2024/05/Agenda-de-Investigac%CC%A7a%CC%83o-em-Sau%CC%81de-Humana-1.pdf.

[17] Universidade Eduardo Mondlane - Historial [Internet]. [cited 2022 February 19]. Available from: https://www.uem.mz/index.php/sobre-a-uem/historial.

[18] Muara J.M.V. (2020). Produção científica em políticas públicas educacionais de Moçambique.

[19] Mattedi M.A., Spiess M.R. (2017). A avaliação da produtividade científica. Hist cienc saude-Manguinhos.

[20] Olaleye D.O., Odaibo G.N., Carney P., Agbaji O., Sagay A.S., Muktar H. (2014). Enhancement of health research capacity in Nigeria through north-south and in-country partnerships. Acad. Med..

[21] Pereira TS, Confraria H, de Lisboa U. Mapeamento da Investigação em Ciências da Saúde. :16.

[22] Pereira et al. - Mapeamento da Investigação em Ciências da Saúde.pdf [Internet]. [cited 2022 October 4]. Available from: https://gulbenkian.pt/wp-content/uploads/2022/09/MAPIS_SE_26_09_2022refB.pdf.

[23] Antunes M. da L., Seguro-de-Carvalho P. (2019). Retrato da produção científica dos Estados-membros da Comunidade de Países de Língua Portuguesa em ciências da saúde: um estudo bibliométrico. Saúde & Tecnologia.

[24] Birkle C., Pendlebury D.A., Schnell J., Adams J. (2020). Web of Science as a data source for research on scientific and scholarly activity. Quantitative Science Studies.

[25] InCites-Indicators-Handbook-6 19.pdf [Internet]. [cited 2023 April 30]. Available from: http://help.prod-incites.com/inCites2Live/8980-TRS/version/default/part/AttachmentData/data/InCites-Indicators-Handbook-6%2019.pdf.

[26] Eck NJ van, Waltman L. (2009). How to normalize cooccurrence data? An analysis of some well-known similarity measures. J. Am. Soc. Inf. Sci. Technol..

[27] van Eck N.J., Waltman L. (2010). Software survey: VOSviewer, a computer program for bibliometric mapping. Scientometrics.

[28] Waltman L., van Eck N.J., Noyons E.C.M. (2010). A unified approach to mapping and clustering of bibliometric networks. Journal of Informetrics.

[29] Langa EA& BN& MPM& FA. MCTES| » Instituições de Ensino Superior [Internet]. [cited 2022 February 6]. Available from: https://www.mctes.gov.mz/instituicoes-de-ensino-superior/.

[30] Andersen KH. Resources and Conflict in Angola. :vol. 87.

[31] História da Universidade Agostinho Neto [Internet]. Universidade Agostinho Neto. [cited 2022 February 19]. Available from: https://uan.ao/historia/.

[32] Cabral F.M.A. (2011).

[33] Vocações para a Matématica Gulbenkian [Internet]. https://joomlakave.com. [cited 2022 February 19]. Available from: https://www.unicv.edu.cv/en/.

[34] D′Abreu I.M.C.V. (2018). http://www.rdpc.uevora.pt/handle/10174/25968.

[35] Raynaud M., Goutaudier V., Louis K., Al-Awadhi S., Dubourg Q., Truchot A. (2021). Impact of the COVID-19 pandemic on publication dynamics and non-COVID-19 research production. BMC Med. Res. Methodol..

[36] Sambo M. do R., Ferreira A.V.L. (2015). Current status on health sciences research productivity pertaining to Angola up to 2014. Health Res. Pol. Syst..

[37] Decreto-57-2017-Redefinicao-INS.pdf [Internet]. [cited 2023 July 21]. Available from: https://ins.gov.mz/wp-content/uploads/2020/09/Decreto-57-2017-Redefinicao-INS.pdf.

[38] Historia-INS.pdf [Internet]. [cited 2023 July 21]. Available from: https://ins.gov.mz/wp-content/uploads/2020/09/Historia-INS.pdf.

[39] de Maio. Diploma Ministerial N° 81/2004.

[40] O INS [Internet]. Instituto Nacional de Saúde. [cited 2023 June 6]. Available from: https://ins.gov.mz/institucional/sobre-o-ins/.

[41] sida3349en-sidas-support-to-the-univerity-eduardo-mondlane-mozambique.pdf [Internet]. [cited 2023 June 19]. Available from: https://cdn.sida.se/publications/files/sida3349en-sidas-support-to-the-univerity-eduardo-mondlane-mozambique.pdf.

[42] Noormahomed E.V., Mocumbi A.O., Ismail M., Carrilho C., Patel S., Nguenha A. (2018). The medical education partnership initiative effect on increasing health professions education and research capacity in Mozambique. Annals of Global Health.

[43] adttscis. 25 years of the Manhiça Health Research Center (CISM) [Internet]. CISM. [cited 2024 July 2]. Available from: https://www.cism25.org/en/home/.

[44] Machele N. (2023). https://www.cismmanhica.org/post/cism-e-isglobal-definem-estratégias-para-os-próximos-5-anos.

[45] PEPFAR - Plano de Emergência do Presidente dos EUA para o Alívio da SIDA - U.S. Embassy in Mozambique [Internet]. [cited 2022 February 19]. Available from: https://mz.usembassy.gov/pt/our-relationship-pt%20/pepfar-us-presidents-emergency-plan-for-aids-relief-pt/.

[46] (2023). Malaria Prevention in Mozambique: Transforming Action into Results | Global Health.

[47] Network Co-Secretariat and Southern Africa Regional Hub Launched at Universidade Eduardo Mondlane [Internet]. NCDI Poverty Network. [cited 2024 July 17]. Available from: https://www.ncdipoverty.org/blog/2021/11/10/mozambique-co-secretariat-launches-at-universidade-eduardo-mondlane.

[48] Instituto Nacional de Saúde. Inquérito Nacional de Prevalência (2022). Riscos Comportamentais e Informação sobre o HIV e SIDA em Moçambique (INSIDA) 2021. Resumo dos principais resultados. https://ins.gov.mz/wpcontent/uploads/2022/12/53059_14_INSIDA_Summary-sheet_POR.pdf.

[49] Ministério da Saúde (MISAU), Instituto Nacional de Estatística (INE) (2015). Inquérito de Indicadores de Imunização, Malária e HIV/SIDA em Moçambique (IMASIDA).

[50] Moçambique Casos de tuberculose, 1960-2022 - knoema.com [Internet]. Knoema. [cited 2023 April 19]. Available from: çhttps://pt.knoema.com//atlas/Moçambique/Casos-de-tuberculose.

[51] Mozambique - Inquérito Nacional sobre Indicadores de Malária (2018). https://microdata.worldbank.org/index.php/catalog/3488.

[52] Agenda-Nacional-de-Pesquisa-em-Saúde_ANAPES.pdf https://ins.gov.mz/wp-content/uploads/2021/03/Agenda-Nacional-de-Pesquisa-em-Sau%CC%81de_ANAPES.pdf.

[53] Strengthening research capacity through the medical education partnership initiative: the Mozambique experience | Human Resources for Health | Full Text [Internet]. [cited 2024 July 17]. Available from: https://human-resources-health.biomedcentral.com/articles/10.1186/1478-4491-11-62. 10.1186/1478-4491-11-62 PMC3895849 24304706

[54] Chavarro D., Tang P., Ràfols I. (2017). Why researchers publish in non-mainstream journals: training, knowledge bridging, and gap filling. Res. Pol..

