# Supplementary material for: Post-independence health research productivity in Portuguese-speaking African countries: A bibliometric analysis of 43 years of research and higher education in Mozambique
Source: Heliyon. 2024 Aug 3;10(15):e35767. doi: 10.1016/j.heliyon.2024.e35767 (PMC11337052; doi:10.1016/j.heliyon.2024.e35767)
Supplement: Multimedia component 1 [file mmc1.docx]

**Supplementary table 1.** Ranking of health science productivity by institution and country, with Universidade Eduardo Mondlane and Instituto Nacional de Saúde segregated.

| N° | Institution | Fr | Country | N° | Institution | Fr | Country |
| --- | --- | --- | --- | --- | --- | --- | --- |
| 1 | Universidade Eduardo Mondlane | 903 | Mozambique | 19 | Vanderbilt University | 78 | United States |
| 2 | Ministério de Saúde | 655 | Mozambique | 20 | University of Pretoria | 76 | South Africa |
| 3 | Centro de Investigação em Saúde de Manhiça | 556 | Mozambique | 21 | Hospital Clínic I Provincial da Universidade De Barcelona | 74 | Spain |
| 4 | Universitat Autònoma de Barcelona | 476 | Spain | 22 | University of Liverpool | 73 | England |
| 5 | Instituto Nacional de Saúde | 269 | Mozambique | 23 | Imperial College London | 66 | England |
| 6 | CDC | 205 | United States | 24 | Harvard University | 64 | United States |
| 7 | Hospital Central de Maputo | 177 | Mozambique | 25 | Swiss Tropical and Public Health Institute | 62 | Switzerland |
| 8 | University of Cape Town | 122 | South Africa | 26 | Universidade Nova de Lisboa | 62 | Portugal |
| 9 | WHO | 118 | World | 27 | Johns Hopkins University | 61 | United States |
| 10 | Hospital Central da Beira | 118 | Mozambique | 28 | Usp – Universidade de São Paulo | 61 | Brazil |
| 11 | University of Washington | 116 | United States | 29 | Universidade Catolica de Moçambique | 60 | Mozambique |
| 12 | Universidade de Porto | 115 | Portugal | 30 | Instituto do Coração | 60 | Mozambique |
| 13 | Université du Witwatersrand | 110 | South Africa | 31 | Instituto de Higiene E Medicina Tropical | 58 | Portugal |
| 14 | Karolinska Institutet | 100 | Sweden | 32 | Friends Global Health | 57 | Global |
| 15 | University of California | 99 | United States | 33 | Aga Khan | 51 | Switzerland |
| 16 | London School of Hygiene & Tropical Medicine | 97 | England | 34 | Medical Research Council (MRC) | 51 | England |
| 17 | Universidade Pedagogica Maputo | 86 | Mozambique | 35 | Universität Tübingen | 50 | Germany |
| 18 | Catalan Institution for Research and Advanced Studies | 80 | Spain | 36 | University of Kwazulu-Natal | 50 | South Africa |

**Note: Fr:** Absolute frequency**, Nº:** Raking.
