# Supplementary material for: Post-independence health research productivity in Portuguese-speaking African countries: A bibliometric analysis of 43 years of research and higher education in Mozambique
Source: Heliyon. 2024 Aug 3;10(15):e35767. doi: 10.1016/j.heliyon.2024.e35767 (PMC11337052; doi:10.1016/j.heliyon.2024.e35767)
Supplement: Multimedia component 2 [file mmc2.docx]

**Supplementary table 2.** The evolution of Private Universities or higher education institutions over time and by province in Mozambique

| Order | University/institution | Abbreviation | Province | Year |
| --- | --- | --- | --- | --- |
|  | Instituto Superior Mãe África | ISMMA | Maputo City | 1992 |
|  | Universidade Politécnica | A POLITÉCNICA | Maputo City | 1995 |
|  | Universidade Católica de Moçambique | UCM | Sofala | 1995 |
|  | Instituto Superior de Ciências e Tecnologias de Moçambique | ISCTEM | Maputo City | 1996 |
|  | Universidade Mussa Bin Bique | *UMB | Nampula | 1998 |
|  | Instituto Superior de Transportes e Comunicações | ISUTC | Maputo City | 2000 |
|  | Universidade Técnica de Moçambique | UDM | Maputo City | 2002 |
|  | Universidade São Tomás de Moçambique | USTM | Maputo City | 2004 |
|  | Universidade Jean Piaget de Moçambique | UJPM | Beira | 2004 |
|  | Escola Superior de Economia e Gestão | ESEG | Maputo City | 2004 |
|  | Instituto Superior de Educação e Tecnologia | ISET | Maputo City | 2005 |
|  | Instituto Superior Cristão | ISC | Tete | 2005 |
|  | Instituto Superior de Formação, Investigação e Ciência | ISFIC | Maputo City | 2005 |
|  | Instituto Superior Dom Bosco | ISDB | Maputo City | 2006 |
|  | Universidade Wutive | UNITIVA | Maputo Province | 2008 |
|  | Instituto Superior Monitor | ISM | Maputo City | 2008 |
|  | Instituto Superior de Comunicação e Imagem | ISCIM | Maputo City | 2008 |
|  | Instituto Superior de Gestão, Comércio e Finanças | ISGECOF | Maputo City | 2009 |
|  | Universidade Alberto Chipande | UNIAC | Sofala | 2009 |
|  | **Instituto Superior de Ciência e Gestão** | INSCIG | Nampula | 2009 |
|  | Universidade Nachingwea | UNA | Maputo Province | 2011 |
|  | Instituto Superior de Gestão de Negócios | ISGN | Maputo Pronvince | 2011 |
|  | Instituto Superior de Estudos e Desenvolvimento Local | ISEDEL | Maputo Province | 2012 |
|  | Instituto Superior Mutasa | ISMU | Manica | 2012 |
|  | Universidade Adventista de Moçambique | UAM | Sofala | 2013 |
|  | Escola Superior de Gestão Corporativa e Social | ESGCS | Maputo City | 2013 |
|  | Universidade Metodista Unida de Moçambique | UMUM | Inhambane | 2014 |
|  | Instituto Superior de Gestão, Administração e Educação | ISG | Maputo City | 2014 |
|  | Instituto Superior de Gestão e Empreendedorismo Gwaza Muthine | ISGE | Maputo Province | 2014 |
|  | Instituto Superior de Ciência de Educação á Distancia | ISCED | Sofala | 2015 |
|  | Instituto Superior de Ciências Empresariais e Tecnológicas | ISCET | Maputo Province | 2016 |
|  | Instituto Superior Politécnico e de Tecnologias | ISPOTEC | Maputo Province | 2018 |
|  | Universidade Áquila | UNAQ | Maputo City | 2018 |
|  | Universidade Técnica Diogo Eugénio Guilande | UTDEG | Maputo Province | 2016 |
